# Technologies for Evaluation of Pelvic Floor Functionality: A Systematic Review

**DOI:** 10.3390/s24124001

**Published:** 2024-06-20

**Authors:** Nikolas Förstl, Ina Adler, Franz Süß, Sebastian Dendorfer

**Affiliations:** 1 OTH Regensburg—Ostbayerische Technische Hochschule Regensburg, Seybothstraße 2, 93053 Regensburg, Germany; ina.adler@oth-regensburg.de (I.A.); sebastian.dendorfer@oth-regensburg.de (S.D.); 2 RCBE—Regensburg Center of Biomedical Engineering, Seybothstraße 2, 93053 Regensburg, Germany

**Keywords:** pelvic floor, sensors, functionality, influence parameters

## Abstract

Pelvic floor dysfunction is a common problem in women and has a negative impact on their quality of life. The aim of this review was to provide a general overview of the current state of technology used to assess pelvic floor functionality. It also provides literature research of the physiological and anatomical factors that correlate with pelvic floor health. This systematic review was conducted according to the PRISMA guidelines. The PubMed, ScienceDirect, Cochrane Library, and IEEE databases were searched for publications on sensor technology for the assessment of pelvic floor functionality. Anatomical and physiological parameters were identified through a manual search. In the systematic review, 114 publications were included. Twelve different sensor technologies were identified. Information on the obtained parameters, sensor position, test activities, and subject characteristics was prepared in tabular form from each publication. A total of 16 anatomical and physiological parameters influencing pelvic floor health were identified in 17 published studies and ranked for their statistical significance. Taken together, this review could serve as a basis for the development of novel sensors which could allow for quantifiable prevention and diagnosis, as well as particularized documentation of rehabilitation processes related to pelvic floor dysfunctions.

## 1. Introduction

Pelvic floor dysfunction (PFD) is a common problem that mainly affects women. The symptoms caused by PFDs negatively affect women’s quality of life [1,2,3]. Women are often restricted in their daily activities due to their symptoms, which can progressively lead to a loss of self-esteem and confidence and further to isolation, frustration, and depression [2]. They even reduce the frequency and intensity of their physical exercising due to their complaints [4]. 

A well-functioning pelvic floor plays an important role in the support and retention of the pelvic organs [5,6], including the bladder, rectum, vagina, and uterus [6]. In addition, a healthy pelvic floor helps to maintain urinary and fecal continence and is essential for a woman’s sexuality and the birth process [5,7]. The female pelvic floor consists of a complex network of muscles, fascia, ligaments, connective tissue, and nerves [5,7,8,9] that is located within the pelvis [10]. Once the integrity of the pelvic floor is compromised, PFDs can occur. The most common PFDs are urinary incontinence (UI), anal incontinence (AI) [11,12], and pelvic organ prolapse (POP) [12,13]. The latter describes a lowering of the pelvic organs.

Nygaard et al. estimated the prevalence of these PFDs in women in the US population. In total, 23.7% of the women studied had at least one PFD, including 15.7% with UI, 9% with fecal incontinence (FI), and 2.9% with POP [14]. Furthermore, the prevalence of PFDs increased progressively with age. Almost half of the women aged 80 years or older suffered from of at least one PFD [14]. As the population ages, the absolute number of PFDs is expected to increase dramatically in the coming years. Therefore, Wu et al. estimated the prevalence of PFD in the US female population from 2010 to 2050. During this period, the number of women with UI will increase from 18.3 to 28.4 million. Huge increases are also expected in the number of women affected by FI and POP. There will be an increase of 59% (from 10.6 million to 16.8 million) for FI and 46% (from 3.3 million to 4.9 million) for POP [15].

There are several methods of assessing pelvic floor functionality and diagnosing PFDs in clinical practice and research. The Oxford Grading Scale (MOS) is currently used to assess the contraction of the pelvic floor muscles [16]. This involves a medical professional palpating the vagina during a pelvic floor contraction and rating the contractility of the pelvic floor muscles on a scale from 0 to 5.

The Pelvic Organ Prolapse Quantification (POP-Q) system is used to objectively assess the severity of pelvic organ prolapse in women [17]. This is a standardized procedure for manually determining anatomical reference points and performing various distance measurements. Measurements are taken during a gynecological examination by medical professionals.

Furthermore, questionnaires on urinary and fecal incontinence (International Consultation on Incontinence Questionnaire Short Form—ICIQ-SF, Fecal Incontinence Severity Index—FISI) are currently used to assess the severity of incontinence [18,19,20]. These include questions about the frequency and severity of symptoms, which are answered by those affected themselves. A scoring system is used to ultimately assess the severity of the incontinence.

In addition to medical examinations and questionnaires, sensor technology is already used in research to assess pelvic floor functionality. Although there are already reviews that provide an overview of sensor technologies related to pelvic floor functionality, these are usually limited to the investigation of one parameter. For example, both Keshwani and McLean and Moser et al. present technologies for measuring pelvic floor muscle activity [21,22], while Liao et al. focus on diagnostic sensors for measuring intra-abdominal pressure (IAP) [23]. However, they do not provide a general overview of all techniques for measuring pelvic floor functionality.

As pelvic floor muscle training (PFMT) is recommended for the treatment of PFDs [24,25], sensor technology is also being used in biofeedback systems. Biofeedback systems are designed to visualize the quality of PFMT and can be used for both prevention and rehabilitation purposes. A review by Woodley et al. presents current technologies designed to support women in performing PFMT [26]. However, they focus more on digital solutions (mobile apps). Again, it does not provide a complete overview of all sensor technologies that may be associated with pelvic floor health.

Despite this range of multifaceted approaches to different focus areas published in the current review literature, most of them specialize only in smaller sub-areas of pelvic floor function sensing technology. The overall aim of this review was to provide a general basis for future research and development with a primary research question on the prevention of PFDs. In particular, this review was divided into a systematic search, with the aim of providing the current state of sensor technology used to assess pelvic floor functionality. In an additional step, a second aim was to gather current knowledge on the physiological and anatomical factors that correlate with PFDs. Altogether, a connection is made between the relevant parameters related to pelvic floor health and the measurable values and technical capabilities.

## 2. Materials and Methods

### 2.1. Study Design

This systematic review was conducted according to the Preferred Reporting Items for Systematic Reviews and Meta-Analysis (PRISMA) guidelines [27]. The submitted pre-print was prospectively registered with the Open Science Framework [28].

### 2.2. Current Sensor Technology Assessing Pelvic Floor Function

#### 2.2.1. Study Identifying

Following an initial search in GoogleScholar, a systematic search of databases was conducted. The electronic databases of PubMed, Cochrane Library, ScienceDirect, and IEEE were searched for publications in the English language. The specific search keywords and strategy with Boolean combinations were “pelvic floor” AND “sensor”. No restrictions were placed on the date of dissemination or the study design. Exceptions were made in the ScienceDirect database. Here, only the most relevant and recent publications from the years 2022 to 2024 were included in the screening process. The relevance of these publications was automatically determined by the database itself based on the number of hits, the significance and occurrence of keywords, proximity, and completeness. After the database research, manual searches of reference lists and citation tracking were undertaken to identify additional articles potentially eligible for inclusion. The search was conducted independently by two researchers in January 2024.

#### 2.2.2. Study Screening

The full screening process was carried out by two researchers, whereas discrepancies were resolved through discussion.

In a first screening, all duplicates were removed, as well as all publications without the implementation of sensors or any evidence on the pelvic floor. Subsequently, the titles and abstracts were screened for the content limits of the review. Excluded were all studies that did not evaluate any specific functions of the pelvic floor. Then, all studies found were screened for eligibility. Inclusion and exclusion criteria were established a priori. 

Studies were excluded based on the following criteria: (1) studies that did not assess pelvic floor function with sensor technology; (2) insufficient methodological quality, including studies with poorly defined research questions, inadequate sample sizes, or insufficient data analysis methods; (3) study designs with only male participants; and (4) studies that did not provide results or conclusions that were directly relevant to the research topic, such as those focusing on outcomes that do not contribute to the relationship between pelvic floor function and sensor technology. To avoid influences from pregnancy and childbirth, studies conducted (5) only with pregnant women or during birth were also excluded. (6) Review articles and case reports referring to specific disease patterns were not included in the analysis.

#### 2.2.3. Study Inclusion

First, the identified studies were sorted according to sensor-type. Data were independently extracted from each study and transferred to a Microsoft Excel file. One spreadsheet per identified sensor-type was conducted. The following data were obtained if available: (1) title; (2) author; (3) year; (4) citation indices from PubMed; (5) sensor type; (6) sensor position; (7) supplement sensor information; (8) secondary sensors used; (9) obtained parameters; (10) reference to pelvic floor; (11) subject characteristics—subject groups, number of subjects, age, and parity; (12) testing position; (13) test activity; (14) and results/discussion/conclusion.

Since the focus of this study is to provide an overview of a wider range of methods, and the studies included therefore differ substantially in some cases, the analysis in this review was limited to a systematic review without a meta-analysis. Due to the high heterogeneity of the studies included in this review, no formal risk assessment using a standardized tool was performed.

### 2.3. Current Knowledge of Physiological and Anatomical Factors Associated with PFDs

#### 2.3.1. Study Identifying

Literature research was conducted to identify physiological and anatomical factors associated with PFDs. This was carried out independently by two researchers using GoogleScholar. The search aimed for studies published in the English language that discuss factors with relations to pelvic floor functionality. There were no restrictions on the date of dissemination, but only studies involving trials with human probands were searched for. Reviews and other descriptive publications were not included. No distinction was made between univariate and multivariate analyses.

#### 2.3.2. Study Screening

The titles and abstracts of the retrieved studies were screened against the content limitations of the search. If the studies screened in the systematic review process mentioned correlations between any type of physiological or anatomical factors affecting pelvic floor health, they were included in the process of this second basis-search.

#### 2.3.3. Study Inclusion

The data were extracted from the identified trials and transferred to a Microsoft Excel file. They were screened for described, identified, discussed, or established physiological or anatomical factors with effects on the pelvic floor functionality—positive or negative. The provided factors were subsequently evaluated according to evidence indicators.

It must be noted that the inclusion of a parameter in the final results table was considered cautiously in each case. If, for example, a study investigated one specific factor relating to the pelvic floor but did not find significant differences in other known factors between its study and control group, those secondary—in this case, nonsignificant—parameters were not included in the results of this review part, as the focus was on some completely different factor.

The rating was illustrated using a color-coded scheme. The evidence was categorized according to the significance indicated in the respective studies. Factors were labelled green when a statistical correlation between a factor and the pelvic floor was identified. For the factors marked in red, no correlation was found.

## 3. Results

The aim of this work was to review the current literature on sensors used in scientific research to assess parameters that provide information on pelvic floor functionality, as well as to obtain physiological and anatomical factors associated with PFDs.

### 3.1. Current Sensor Technology Assessing Pelvic Floor Function

**Figure 1:**
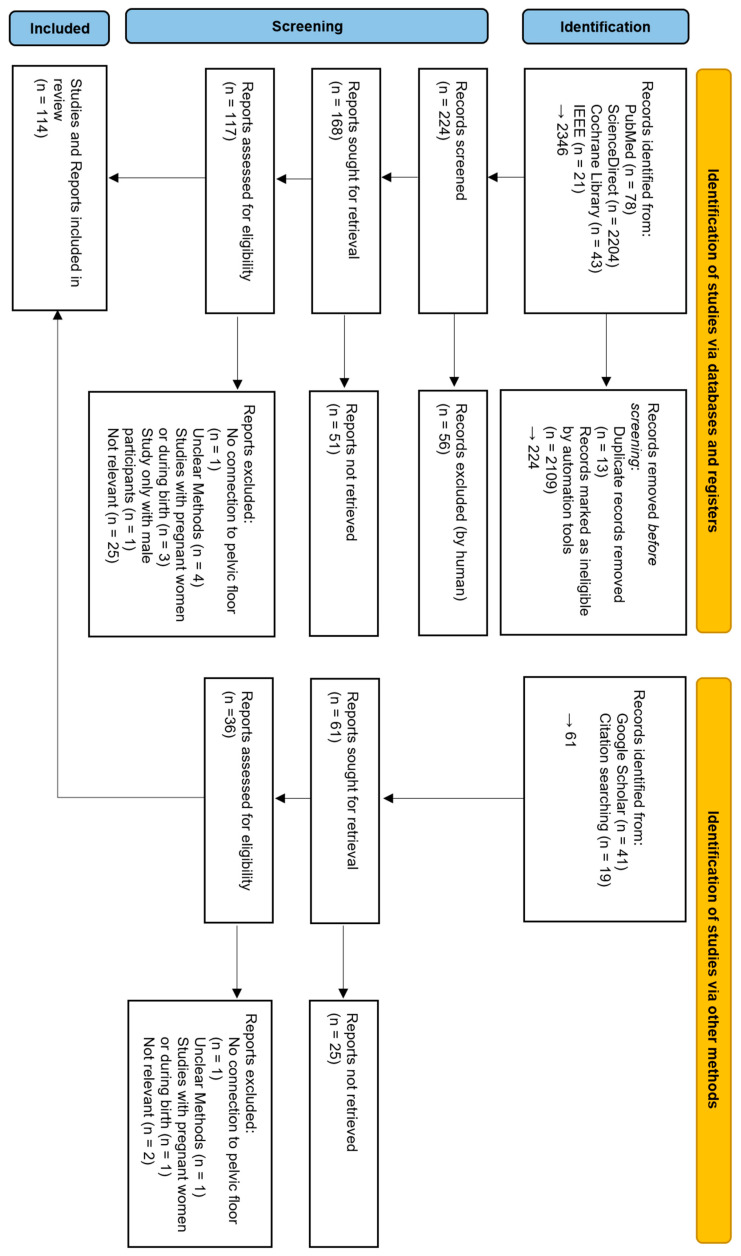
PRISMA flow diagram of the study-selection process.

The flowchart in Figure 1 summarizes the process of study selection. An initial GoogleScholar search identified 41 records. The identification of studies via databases and registers concluded in a total of 2346 records, of which 2121 publications were removed before screening. A further citation search identified an additional 19 studies.

A total of 286 records were screened. In this screening process, in summary, 76 studies were excluded after a preliminary analysis of the title and abstract. During the assessment for eligibility, 39 records were excluded, as they fulfilled at least one of the exclusion criteria. This results in a total of 114 studies and reports included in this review.

**Figure 2:**
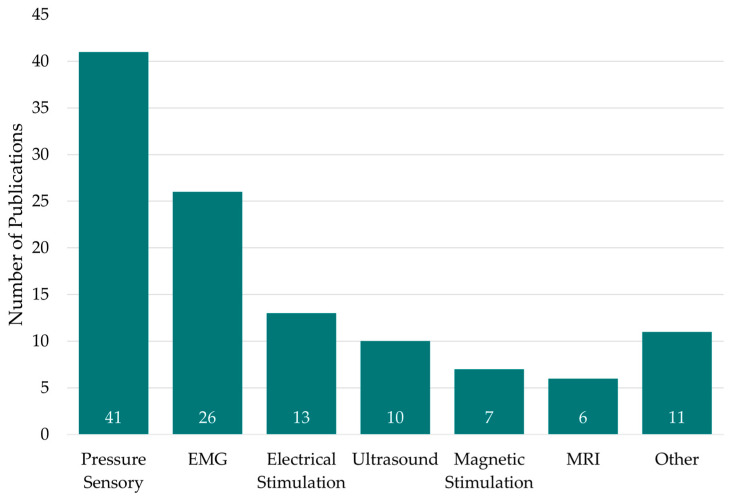
Number of publications included in this review, presented by their corresponding sensor type.

The sorting of the included records resulted in 12 different sensor types: accelerometer, electrical stimulation (ES), electromagnetic tracking (EMT), electromyography (EMG), magnetic stimulation (MS), magnetic resonance imaging (MRI), photogrammetry, pressure sensors, ultrasound, vibration, X-ray, and infrared thermography (IRT). No distinction was made between the type of application of each sensor. Therefore, sensors for diagnostic, therapeutic, and preventive applications were included in this review. Figure 2 shows the distribution of the publications included in the review, along with the respective number of studies identified per sensor category. The largest number of publications was found utilizing pressure sensors (41 studies) and EMG sensors (26 studies). Accelerometer (two studies), EMT (two studies), photogrammetry (two studies), vibration (one study), X-ray (three studies), and IRT (one study) were grouped as “Others” due to their much lower number of occurrences.

The extraction of the relevant data from the 114 studies resulted in a tabular file containing 12 tables, 1 per identified sensor-type, each with 14 columns. These are provided in the Supplementary Materials. For the purpose of simplification, an excerpt from the extensive table is shown in Table 1, Table 2, Table 3, Table 4, Table 5, Table 6 and Table 7, containing the following data for each sensor type: (1) author, year; (2) obtained parameter; (3) sensor position; (4) test activity; and (5) subject characteristics—subject groups, number of subjects, and age. For ES and MS, the data categories are slightly adapted. Since no parameters are determined by these technologies, but they are used to stimulate the pelvic floor muscles, the extracted data include (1) author, year; (2) stimulated part; (3) sensor position; (4) stimulation type; and (5) subject characteristics—subject groups, number of subjects, and age.

**Figure 3:**
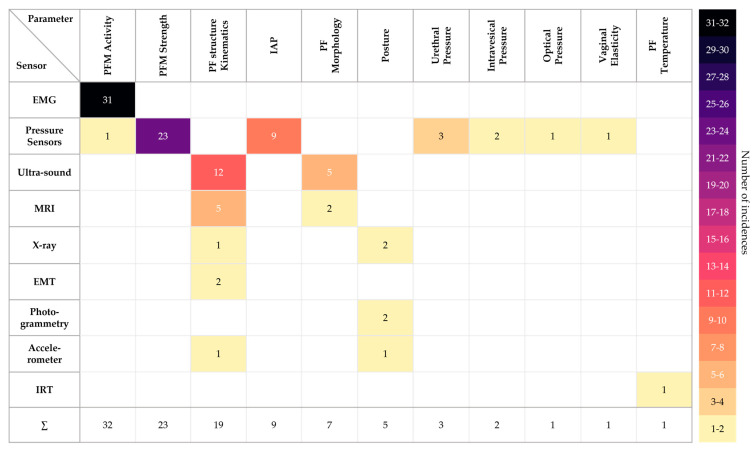
Obtained Parameters with the extracted sensors with their number of acquirement.

Figure 3 provides an overview of all parameters observed in the included publications, highlighting which sensors recorded each parameter and how many times it was acquired. Higher-level categories were used to group as many specific parameters as possible. Thus, among others, coccyx movement, urethra kinematics, puborectal position, and intravaginal acceleration were categorized as PF structure kinematics. Similarly, for example, thoracic kyphosis and lumbar lordosis were summarized as posture. When PFM activity was included, all sensor application types were grouped together and no longer distinguished between, for example, vaginal, rectal, perirectal, or other approaches. Similarly, vaginal, and rectal measurement methods were combined for the PFM strength parameter. Of all the parameters identified for all the sensors, PFM activity (32 times), PFM strength (23 times), and pelvic floor structure kinematics (19 times) were obtained the most. Only in a few cases were optical pressure, vaginal elasticity, and pelvic floor temperature (one time each) measured.

**Figure 4:**
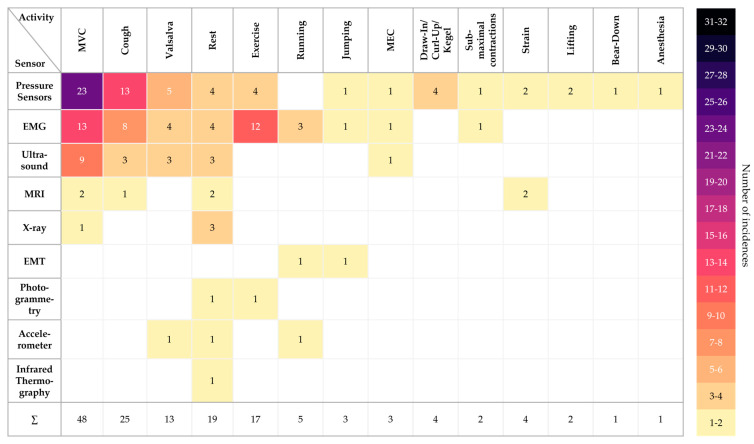
Activities performed during the data assessment with the respective sensors, along with their number of occurrences.

Figure 4 shows which activities were included in how many of the papers and how many times they were recorded. Of all 12 sensor types, maximal voluntary contraction (MVC) was by far the most recorded, with a total of 48 times. Activities such as jumping (three times), straining (four times), or lifting (two times) were recorded much less frequently in all the studies.

ES, MS, and vibration are not listed in Figure 3 and Figure 4, as these are sensors that do not measure anything per se but have been investigated as therapeutic or preventive tools.

### 3.2. Current Knowledge of Physiological and Anatomical Factors Associated with PFDs

The search for physiological and anatomical factors associated with PFDs resulted in the inclusion of 17 studies published between 1994 and 2023. The search identified 16 influencing parameters: age, menopause, parity, type of delivery, fetal macrosomia, hormone therapy, BMI, race, chronic cough, hysterectomy, physical activity, gastrointestinal pathology, gynecological pathology, other diseases (arthritis or osteoporosis), respiration, and spine curvature. Each study examined between one and ten of the factors listed. Table 8 summarizes the results obtained in this part of the review. The parameters age and parity were analyzed in nine of the included studies each. They are therefore the most common factors in the listed publications, followed by BMI (seven times), mode of delivery (six times), and spinal curvature (five times). The color scheme classifies each assessed parameter according to the level of evidence found in the respective study. Green indicates statistical correlation found, and red indicates no correlation found. When accumulated, nine factors were rated green, and seven were rated red. The parameters labelled green include, for example, age, parity, and BMI. In contrast, mode of delivery, hormone therapy, and fetal macrosomia are among the parameters marked in red.

## 4. Discussion

The aim of this work was to provide an overview of the current state of technology used to assess pelvic floor functionality, as well as of the current knowledge of the physiological and anatomical factors that correlate with PFDs.

In combination, these two reviews aim to provide an overview of which functional parameters of the pelvic floor are already covered by sensor technology and for which there is still potential for expansion. This information will add value to the development of future pelvic floor sensor technologies.

### 4.1. Current Sensor Technology Assessing Pelvic Floor Functionality

#### 4.1.1. Distribution of the Sensor Technologies in the Included Publications

Figure 2 shows the distribution of the publications included in the review with the respective number of studies identified per sensor category. However, this should be seen only as a quantitative comparison. The figure reflects only how often each sensor type was used in the studies to assess pelvic floor functionality. Neither the relevance nor the evidence of a particular sensor technology for assessing pelvic floor functionality can be automatically inferred from their frequency of appearance in the table. Nevertheless, the frequent use of certain sensor types indicates that this technology has become somewhat established in the assessment of pelvic floor functionality.

The most commonly used sensor technologies, based on the studies identified, include pressure sensors, EMG, and ES. As the review considers sensors for prevention, diagnosis and rehabilitation, the identified sensor categories can be further broken down into their application areas. For diagnostic purposes, the technologies used are pressure measurement, EMG, ultrasound, MRI, accelerometry, EMT, photogrammetry, X-ray, and IRT. Pressure and EMG measurements are also used in biofeedback systems for prevention and rehabilitation purposes. In addition, ES, MS, and vibration are used for therapeutic applications. Categorizing sensors according to their application can serve as a support for the development of new technology or methods, for example, by extending the areas of application for existing sensors. Depending on the future purpose of a new sensor, different established technologies can be used as a reference.

#### 4.1.2. Data Extracted from the Included Publications

All technologies included in the identified studies were categorized into different sensor types. The information from the studies was tabulated into different data categories. Identical data categories were used for most sensor types (Table 1, Table 2, Table 4, Table 6 and Table 7). The results of these sensor types are therefore comparable. For the sensor types ES (Table 3) and MS (Table 5), the data categories were slightly adjusted, as these technologies do not provide parameters but are used to stimulate PFM. The results of these technologies can be compared with each other. However, the comparison of sensor types from Table 1, Table 2, Table 4, Table 5 and Table 7 with the ones listed in Table 3 and Table 5 should be made with caution due to their different mechanism of action.

#### 4.1.3. Measured Parameters

Figure 3 provides an overview of all the parameters relating to the female pelvic floor that have already been measured by sensors. The parameters listed contribute to the assessment of pelvic floor functionality. In particular, the PFM activity parameter is extensively summarized in Figure 3 and no longer differentiates between vaginal, rectal, or perirectal approaches. However, more detailed information can be found in the corresponding table, under “Sensor Position”. The color scheme in the tables shows which parameters have been measured with which sensor in how many publications. This reflects only the number of times a measurement method has been used to determine a particular parameter. The relevance and evidence of a particular parameter for assessing pelvic floor functionality should not be equated with the number of studies found for that parameter.

New, innovative methods for measuring other parameters related to pelvic floor functionality may provide relevance in the future, even if they have not been studied much to date. For example, the IRT method for measuring pelvic floor temperature [142] (published 2022) and an optical method of measuring pressure [61] (published 2023) were each found in only one study. However, this does not automatically mean that these new measurement methods and the parameters they provide are any less relevant or accurate.

The parameters obtained from EMG and pressure measurements are very different from those obtained from the other sensor types. These technologies are the most widely used in the current literature. It is therefore reasonable to analyze them in more detail.

EMG measurements were used to measure the activity of the PFM. Most studies used invasive vaginal and rectal surface EMG probes, while the remaining studies used surface EMG sensors around the perineum and anus. As the sensors are placed in a very intimate part of the body, the measurements may cause discomfort and embarrassment to the women. In addition, surface EMG measurements are often prone to error, so their results should be interpreted with caution. Identical positioning of the intravaginal and rectal EMG probes is a prerequisite for comparing results within a group of different women and within one test person (e.g., different trails and different training conditions). Furthermore, crosstalk is to be expected when using surface EMG measurements. If other surrounding muscles are activated in addition to the muscle area under consideration, this may have a negative effect on the validity of the measurement results. Especially in surface EMG measurements of pelvic floor activity during highly dynamic movements, crosstalk cannot be ruled out. Both Moser et al. and Leitner et al., who performed intravaginal surface EMG measurements during jumping and running, mentioned the possibility of crosstalk as a limitation of their studies [80,81]. Despite the considerable potential for error of the EMG method, it currently appears to be the most effective method for measuring muscle activity in the pelvic floor.

Pressure sensors are mainly used in the literature to determine intravaginal and rectal PFM strength. They are also used to measure intra-abdominal, urethral, and intravesical pressures. It is therefore currently possible to measure both the pressure acting on the pelvic floor (IAP) and the pressure in the vagina/rectum caused by a contraction of the pelvic floor muscles (PFM strength). As these are mainly invasive measurements, there may be limitations to the types of activity that can be recorded by these techniques. Nevertheless, the current techniques for determining various pressure parameters should be considered as a basis for the further development of sensors.

Despite the limitations, EMG and pressure sensors have convincing advantages due to their simple handling, easy accessibility, and practical and versatile application. This makes them suitable for everyday use, as well as for conducting scientific studies.

#### 4.1.4. Measured Activities

Figure 4 shows which types of activity have already been measured with which sensor technology. The color scheme for the different sensor–activity combinations merely illustrates the number of studies identified; thus, it should not automatically be equated with the relevance of a sensor technology for measuring a particular activity.

All types of activities listed in Figure 4 were observed for their activation of the pelvic floor muscles. However, depending on the type of activity, there is either a conscious or unconscious contraction of the pelvic floor muscles. In activities such as MVC, draw-in/curl-up/Kegel, sub-maximal contraction, and MEC, muscle activation is voluntarily induced. In contrast, involuntary activity in the pelvic floor muscles is thought to occur during coughing, exercising, running, jumping, straining, lifting, and performing the Valsalva and bear-down maneuvers. This difference should be kept in mind when developing future sensors. Depending on the application, different sensor technologies should be considered as a basis. For example, when measuring high-activity movements, the sensor technology needs to be able to adapt to these movements; for example, X-ray or MRI are likely to be unsuitable for dynamic recordings.

#### 4.1.5. Future Sensor Developments

In the early stages of developing a new, innovative tool, this overview can be used to categorize technologies and parameters according to your own predefined requirements. Table 1, Table 2, Table 3, Table 4, Table 5, Table 6 and Table 7 can be used to eliminate inconvenient sensors and technologies and to compare all the requirement criteria against the tables to find a suitable technology. An example of a specific field of application for this would be the ever-increasing development of AI-supported sensor technology. The findings from the existing technology can be taken as a starting point. The overview from this review simplifies this process.

Table 1, Table 2, Table 3, Table 4, Table 5, Table 6 and Table 7 could be used to exclude and include sensor technology, depending on the invasiveness, type of movement patterns, static or dynamic measurements, recording of voluntary or involuntary contractions, or the location of use (home or practice).Therefore, if the main focus of the sensor development is put on a non-invasive sensor, all sensors with the positioning stated as intravaginal or intrarectal cannot be taken into consideration. Likewise, the use of MRI and X-ray is not suitable for a device intended to be used as a home-application tool.

In addition, with the information gained from this review, the further advancement of existing technologies can be taken in a new direction. The tables in the Section 3 of this work show the different application areas of 12 different sensor types. This allows developers to find impulses for individual extensions of their own modules. Ideas can be found in the comparison of one’s own sensor group, as well as in the application areas of another sensor group and thus positively enrich innovative sensor development.

### 4.2. Current Knowledge of Physiological and Anatomical Factors Associated with PFDs

What is missing from the list of physiological and anatomical factors affecting the pelvic floor is an assessment of the interactions between the individual factors. For example, it is known that the stage of a woman’s menopause is strongly linked to her age [154]. Diseases such as osteoporosis and arthritis also occur more frequently at an older age [155,156]. Now, further studies are needed to concretize the correlations between the parameters for the particular application area of pelvic floor health. For some factors, it is also necessary to further investigate whether they are responsible for the pelvic floor dysfunctions or whether a change in a parameter is a consequence of PFDs. An example would be the spinal curvature parameter.

A closer look at Table 8 reveals that the results of the 17 sources considered are sometimes not entirely consistent. For example, in the case of age and BMI, all the sources agree on a significant influence. The situation is more differentiated when it comes to the Mode of Delivery, for example. Three of the sources found a significant association with pelvic floor health, and three found no significant association. For such controversial parameters, a more detailed analysis and comparison of publications is needed for future work. Nevertheless, in most cases, the table provides a good and clear initial overview of the parameters considered in relation to pelvic floor health. With the exception of the abovementioned example, the color differentiation allows for a relatively clear tendency to be identified in terms of the determined significance.

### 4.3. Discussion of the Combination of All Results

A closer look at the parameters found in the second literature search suggests that some of the factors are partly biologically predetermined. However, DeLancey et al. emphasize in their study that the functionality of the pelvic floor can be actively influenced, both positively and negatively, by self-imposed variables [157]. The biological changes that occur, in particular, with increasing age do not preclude pelvic floor health. Combining this knowledge with sensors that can objectively assess the condition of the pelvic floor offers great opportunities to make a lasting—and positive—difference in the lives of many women.

This point can, in turn, influence both the development of new sensor systems and the expansion of existing systems. With the background knowledge of which parameters have an influence on pelvic floor health, the development of new systems for parameters that are not currently measured can be initiated. Likewise, existing systems can be expanded to include factors that can possibly be recorded with the existing system but have not yet been integrated as output parameters.

### 4.4. Limitations

For the systematic review, there were a few restrictions on the inclusion criteria in order to cover all existing sensor technologies. Therefore, a large number of publications had to be considered. Subject studies that had already used established measurement technology were included. Other publications investigating the development of new sensor prototypes were also considered. Due to the different nature of the studies, it was not possible to compare them, and therefore no meta-study was conducted.

The second literature search was an initial, non-structured search, which resulted in a listing of studies. Therefore, the results may not be entirely representative of the current state of the art. More detailed studies need to be carried out to provide specific information on the parameters and to what extent each factor influences pelvic floor health and in what way each parameter has a positive or negative effect.

When evaluating the evidence of the individual parameters on the basis of the statistical assessment in the respective publications, it must be noted that different statistical tests and significance criteria were partially used. This was not taken into account in the list in Table 8. The color coding was based solely on the significance reported in the publications. The statistical methods and criteria used were disregarded.

## 5. Conclusions

The presented review that combines existing sensor technologies for assessing pelvic floor functionality with relevant anatomical and physiological parameters related to the pelvic floor could serve as a basis for further research. The field of sensor technology development can benefit from this work. Possible combinations of different sensor technologies could be considered to improve the functionality and acceptability of sensor applications in the field of pelvic floor health. 

Identifying the physiological and anatomical factors involved in pelvic floor disorders provides a fundamental basis for the further expansion of technology integration in healthcare. Combined with further research and development of sensor technology that addresses these relevant factors, this work offers opportunities to facilitate the reference to diagnostic, prevention, and rehabilitation tools for PFDs.

Specifically, this involves quantifiable prevention and diagnosis, as well as detailed documentation of rehabilitation processes related to PFD. Taken together, this work highlights the opportunities for the development of novel sensors, as well as for their further optimization for the diagnosis, prevention, and rehabilitation of PFDs.

## Figures and Tables

**Table 1:** Data extracted from the reviewed studies including pressure sensors.

Study	Obtained Parameter	Sensor Position	Test Activity	Subject Characteristics		
				Group	Number	Mean Age (Years)
**Pressure Sensor/Transducer/Force Sensor**						
Shaw et al., 2014 [29]	IAP	Intravaginal	31 activities of different intensity	Women	57	30.4 ± 9.3
Dietze-Hermosa et al., 2020 [30]	IAP	Intravaginal	31 activities of different intensity	Women	57	30.4 ± 9.3
Niederauer et al., 2017 [31]	IAP	Intravaginal	-	-	-	-
Rosenbluth et al., 2010 [32]	IAP	Intravaginal	Coughing, Valsalva, squatting and jumping	Women undergoing routine filling cystometry	-	<21
Djivoh and Jaeger, 2023 [33]	IAP, PP	Intrarectal and perineal	Coughing, MVC, curl-up, diaphragmatic aspiration, drawing-in	Postpartum women	17	28.5 ± 4.7
Parkinson et al., 2019 [34]	Vaginal elasticity	Intravaginal	Valsalva, coughing, MVC	Parous women: Group 1: colposcopy Group 2: INC	Group 1: 6 Group 2: 4	18–76
Tian et al., 2018 [35]	IAP	Intravaginal	10 exercises	Women	53	39
Nygaard et al., 2021 [36]	IAP	Intravaginal	Lifting a car seat	Postpartum women	593	29.6 years ± 5.0
de Abreu et al., 2019 [37]	Lower abdominal MA	Under the abdomen	Drawing-in	Women with low back pain: Group 1: CON Group 2: INC	54 Group 1: 23 Group 2: 31	Group 1: 54.6 ± 8.0 Group 2: 50.5 ± 7.5
Constantinou and Omata, 2007 [38]	Distribution of anisotropic forces acting on the vagina	Intravaginal	MVC, coughing in supine	Women	6	63.8 ± 9.8 years
Constantinou et al., 2007 [39]	Distribution of anisotropic forces acting on the vagina	Intravaginal	MVC, coughing in supine	Women	6	63.8 ± 9.8 years
Hsu et al., 2018 [40]	IAP	Intravaginal	Lifting a car seat	Postpartum women	206	27.38 ± 5.00
El-Hamamsy et al., 2021 [41]	IAP	Intrarectal	Valsalva, bear down in supine and standing	Group 1: healthy men Group 2: healthy nulliparous women	Group 1: 10 Group 2: 10	25 ± 9.25
Tan-Kim et al., 2010 [42]	Intravesical and urethral pressure	Intravesical and urethral	Valsalva, coughing in supine and standing	Women: Group 1: CG Group 2: INC	Group 1: 18 Group 2: 7	Group 1: 40 Group 2: 65
Omata et al., 2003 [43]	PFM strength	Intravaginal	MVC, coughing	-	-	-
Cacciari et al., 2017 [44]	PFM strength	Intravaginal	MVC, Valsalva	Women	26	37.0 ± 10.8
Peng et al., 2007 [45]	PFM strength	Intravaginal	Rest, MVC in supine	Women: Group 1: CG Group 2: INC	Group 1: 23 Group 2:10	Group 1: 39.0 ± 2.3 Group 2: 51.5 ± 5.3
Horng et al., 2022 [46]	PFM contraction strength and duration	Between the upper inner thighs	Kegel exercises	Women, INC	60	53 ± 5.53
Paasch et al., 2023 [47]	-	Sit on a sensor, not inserted	PFMT—Kegel exercises	INC: Group 1: PFMT + sensor Group 2: CG	22 women, 2 men: Group 1: 10 Group 2: 14	Group 1: 42.5 Group 2: 41.0
van Raalte and Egorov, 2015 [48]	Vaginal pressure—responses from the vaginal walls	Intravaginal	Vaginal Tactile Imager (VTI) examination in supine	Women: Group 1: CG Group 2: POP	20 Group 1: 4 Group 2: 16	Range: 41 to 70
Lee et al., 2013 [49]	PFM strength	Extracorporeal; Chair	MVC, EVC	Women, INC	71	52.2
**Fiber Optic Pressure Sensor**						
Stafford et al., 2020 [50]	urethral pressure	Intravesical	sub-maximal contractions, MVC, coughing	Group 1: woman Group 2: man	Group 1: 1 Group 2: 1	Group 1: 33 Group 2: 47
Parkinson et al., 2022 [51]	intravaginal pressure	Intravaginal	MVC, automated probe dilation cycle	Group 1: MVC measurements Group 2: resting tissue resistance measurements	Group 1: 46 Group 2: 19	Group 1: 47.8 ± 20.0 Group 2: 45.0 ± 21.5
Smith, et al., 2000 [52]	PFM strength	Intravaginal	-	Women, INC	44	54
**femfit^®^ (array of eight pressure sensors)**						
Marriott et al., 2021 [53]	intravaginal pressure	Intravaginal	Rest, MVC, coughing	Women with vaginal and/or uterine prolapse	19	63 ± 11.1
Kruger et al., 2019 [54]	Intravaginal pressure	Intravaginal	MVC, MVC of abdominal and hip muscles, coughing	Women	21	43.7 ± 11.3
Pedofsky et al., 2019 [55]	Intravaginal pressure	Intravaginal	Rest, MVC, coughing in supine and standing	Women with a vaginal/uterine prolapse	10	-
**Manometer**						
Lambert et al., 2005 [56]	Bladder pressure/IAP	Intravesical	Under anesthesia	Group 1: obese women Group 2: obese man Group 3: CG	55 Group 1: 37 Group 2: 8 Group 3: 4	Group 1 + 2: 38 ± 2 Group 3: 46 ± 5
Fitz et al., 2017 [57]	PFM strength	Intravaginal	MVC, training exercises in supine, sitting and standing	Women, INC: Group 1: manometry-BF Group 2: PFMT	49 Group 1: 25 Group 2: 24	Group 1: 56.1 ± 10.5 Group 2: 56.6 ± 12.0
Colombage et al., 2023 [58]	PFM strength	Intravaginal	MVC	Group 1: women with breast cancer with treatment Group 2: CG	32 women Group 1: 16 Group 2: 16	Group 1: 41.4 ± 7.3 Group 2: 42.2 ± 7.6
Attari et al., 2020 [59]	Intrarectal and anal sphincter pressure	Intrarectal	Rest, MVC, simulated defecation	Previous anorectal or colonic surgery; men and women	16	median age: 61
Kirby et al., 2015 [60]	Maximum urethral closure pressures	Intraurethral	Cough and strain conditions	Women, INC	65	-
**Optical Pressure Sensing Array**						
Newcombe et al., 2023 [61]	PFM pressure: change in the optical attenuation of light passing through a material that is being compressed by an unknown pressure.	To develop	-	-	-	-
**Perineometer**						
Celiker Tosun et al., 2015 [62]	PFM strength	Intravaginal	MVC	Women, INC Group 1: PFMT Group 2: CG	Group 1: 65 Group 2: 65	Group 1: 51.7 ± 10.3 Group 2: 52.5 ± 9.1
Hwang. et al., 2021 [63]	PFM function: strength and endurance	Intravaginal	MVC	Women, INC	42	42.9 ± 8.1
de Oliveira et al., 2016 [64]	PFM strength	Intravaginal	MVC	Women: Group 1: waist < 80 cm Group 2: waist > 80 cm	Group 1: 70 Group 2: 86	Group 1: 55.21 ± 5.24 Group 2: 57.23 ± 6.12
Peschers et al., 1997 [65]	PFM strength	Intravaginal	MVC	Women: Group 1: primipara Group 2: multipara Group 3: CG: caesarean delivery	55 Group 1: 25 Group 2: 20 Group 3: 10	Group 1: 28.2 ± 4.31 Group 2: 31.9 ± 3.88 Group 3: 30.2 ± 4.9
**Dynamometer**						
El-Sayegh et al., 2020 [66]	PFM forces	Intravaginal	-	-	-	-
Niederauer et al., 2019 [67]	Vaginal closure force	Intravaginal	-	-	-	-
Chamochumbi et al., 2012 [68]	Maximal vaginal aperture; PFM active and passive strength	Intravaginal	MVC in supine	Women: Group 1: CG Group 2: INC	Group 1: 16 Group 2: 16	Group 1: 37 ± 8 Group 2: 48 ± 7
Romero-Cullerés et al., 2017 [69]	PFM strength	Intravaginal	MVC in supine	Women, INC	102	56 ± 10.3

**Table 2:** Data extracted from the reviewed studies including EMG sensors.

Study	Obtained Parameter	Sensor Position	Test Activity	Subject Characteristics		
				Group	Number	Mean Age (Years)
**Surface EMG**						
Alves et al., 2015 [70]	PFM activity	Intravaginal	MVC in supine position	Women; Group 1: INC Group 2: CG	Group 1: 18 Group 2: 12	INC: 66.11 ± 8.72 CG: 65.67 ± 9.21
Albaladejo-Belmonte et al., 2021 [71]	PFM activity	Perineum	MVC in dorsal lithotomy position	Women; Group 1: >35, P Group 2: <35, NP Group 3: >18, CPP	Group 1: 24 Group 2: 24 Group 3: 24	Group 1: 40.9 ± 7.2 Group 2: 28.1 ± 3.2 Group 3: 43.8 ± 8.8
Albaladejo-Belmonte M. et al., 2021 [72]	PFM activity	Electrodes on the Labia Majora	Relaxed state, MVC in dorsal lithotomy position	Women; Group 1: CPP Group 2: healthy	Group 1: 24 Group 2: 24	Group 1: 43.8 ± 8.8 Group 2: 40.9 ± 7.2
Bertotto et al., 2017 [73]	PFM activity	Intravaginal	MVC, coughs; lithotomy, supine, seated, and standing positions	postmenopausal women with INC Group 1: CG Group 2: PFME Group 3: PFME + BF	Group 1: 14 Group 2: 15 Group 3: 16	Group l: 57.1 ± 5.3 Group 2: 59.3 ± 4.9 Group 3: 58.4 ± 6.8
Kannan et al., 2022 [74]	PFM activity	Perineum	PFMT in lying, sitting and standing positions	Women INC; Group 1: PFMT with PelviSense Group 2: PFMT with BF Group 3: CG	Group 1: 17 Group 2: 17 Group 3: 17	Group 1: 49.3 ± 5.5 Group 2: 52.5 ± 6.2 Group 3: 46.8 ± 8.3
Chmielewska et al., 2019 [75]	PFM activity	Endovaginal	MVC in supine position, sEMG during 5 exercises in lying supine and standing position	Women; Group 1: PFMT with BF Group 2: Training with Pilates	Group 1: 18 Group 2: 13	Group 1: 52.9 ± 4 Group 2: 51.5–6 ± 5.2
Capelini et al., 2006 [76]	PFM activity	Intravaginal	PFMT in lithotomy position	Women, INC;	14	49.6
Hirakawa et al., 2013 [77]	PFM activity	Intravaginal	PFMT	Women, INC; Group 1: PFMT Group 2: PFMT with BF	Group 1: 23 Group 2: 23	Group 1: 58.3 ± 11.2 Group 2: 55.3 ± 9.8
Blagg and Bolgla, 2023 [78]	PFM activity (levator ani)	Perianal	Yoga poses	healthy, NP, regularly exercising females	25	23.7 ± 2.2
Voorham-van der Zalm et al., 2013 [79]	PFM activity	Intravaginal, Intrarectal	MVC, MEC, coughs, Valsalva maneuvers in supine (women) or side (men) position	Group 1: males Women: Group 2: NP, premenopausal Group 3: P, premenopausal Group 4: NP, postmenopausal Group 5: P, postmenopausal	Group 1: 61 Group 2: 86 Group 3: 37 Group 4: 5 Group 5: 40	Group 1: 41 (19–70) Group 2: 24 (18–49) Group 3: 44 (32–56) Group 4: 54 (50–65) Group 5: 58 (51–72)
Moser et al., 2018 [80]	PFM activity	Intravaginal	CMJ, DJ	Women; Group 1: CON Group 2: INC	Group 1: 28 Group 2: 22	21–58
Leitner et al., 2016 [81]	PFM activity	Intravaginal	Running at 7, 11, and 15 km/h	Women; Group 1: CON Group 2: INC	Group 1: 28 Group 2: 22	18–60
Hodges et al., 2007 [82]	PFM activity, Activity of external anal sphincter (men)	Women: Intravaginal Men: Intrarectal	Rapid arm movements, different types of breathing, in standing position	Women; Men;	Women: 6 Man: 1	Women: 45.7 (35–63) Man: 30
Koenig et al., 2020 [83]	PFM activity, wavelet analyses	Intravaginal	Running 7, 11, and 15 km/h	Women; Group 1: CON Group 2: INC	Group 1: 28 Group 2: 21	18–60
Chen et al., 2005 [84]	PFM activity	Intravaginal	PFM rest and MVC standing, standing with the ankles dorsiflexed and plantar flexed	Women with INC;	39	38–72
Junginger et al., 2018 [85]	PFM activity	Intravaginal	Maximal and submaximal PFMC in upright position	Women; Group 1: CON Group 2: INC	Group 1: 14 Group 2: 68	28–77 (median age 47)
Lee et al., 2019 [86]	PFM activity	Intravaginal, Intrarectal	Ankle dorsiflexion and plantar flexion in standing position and in long sitting position	healthy adults (Women, Men) without pelvic floor dysfunction	Women: 33 Men: 28	Women: 43.04 Men: 37.86
Navarro Brazález et al., 2020 [87]	PFM activity	Perineum	Hypopressive exercise in supine position with one leg raised, then in an orthostatic position	Parous women;	66	45
Chmielewska et al., 2015 [88]	PFM activity	Intravaginal	MVC in lying, sitting, standing position	Healthy nulliparous women;	20	19–28
**Surface EMG-Periform**						
Sapsford and Hodges, 2001 [89]	MA of pubococcygeus	Women: Intravaginal Men: Intrarectal	MVC; AMM in supine and standing position	Women; Man;	Women: 6 Man: 1	Women: 45.7 (35–63) Man: 30
Capson et al., 2011 [90]	PFM activity	Intravaginal	standing, coughing, Valsalva, MVC; load-catching task in three different standing postures	Women; NP	16	Between 22 and 41
García-Arrabé et al., 2023 [91]	PFM activity	Intravaginal	Running at 9, 11, and 13 km/h with two types of shoes	Female recreational runners, NP	10	20–38
Smith et al., 2006 [92]	PFM activity	Intravaginal	Flex/extend their right arm as fast as possible in standing position	Women; Group 1: CON Group 2: INC	Group 1: 14 Group 2: 16	Group 1: 52.5 Group 2: 49.8
Sapsford et al., 2001 [93]	PFM activity	Intravaginal	MVC, lying with hips flexed to 60°, three different lumbar spine positions	Parous women;	7	49.3 (39–64)
**Needle EMG**						
Deindl et al., 1993 [94]	MA of pubococcygeal muscles	Percutaneously into pubococcygeal muscles	MA of pubococcygeal muscles during relaxation, MVC, squeezing, cough, Valsalva in supine and erect position	women; NP, CON	10	22–32
Shafik et al., 1991 [95]	MA of puborectalis muscle	Into puborectalis muscle	Coughing or Valsalva’s maneuver	Women; Men;	Women: 9 Men: 10	38.6 (22–58)

**Table 3:** Data extracted from the reviewed studies including Electrical Stimulation.

Study	Stimulated Part	Sensor Position	Stimulation Type	Subject Characteristics		
				Group	Number	Mean Age (Years)
**ES**						
Barroso Jr. et al., 2014 [96]	Urethral external sphincter muscle	Perineum	During sleeping	Nocturnal enuresis patients	6	11
Frazén et al., 2010 [97]	PFM	Vaginally and/or transanally	20 min stimulation sessions; 1–2 times per week	Women; INC Group 1: ES Group 2: tolterodine receive	61 Group 1: 31 Group 2: 30	Group 1: 55 Group 2: 61
Wang and Zhang, 2012 [98]	Pudendal nerve—urethral external sphincter	Pudendal nerve—lower back	60 min stimulation sessions, 3 times per week	Women, INC 3 groups with different doctors	Group 1: 35 Group 2: 60 Group 3: 30	Group 1: 54.9 ± 9.7 Group 2: 55.0 ± 10.6 Group 3: 57.9 ± 10.6
Hwang et al., 2021 [99]	PFM	Sacral and perivaginal regions—sitting on device	15 min stimulation sessions, 5 times per week	Women, INC Group 1: ES Group 2: CG	Group 1: 17 Group 2: 16	Group1: 42.1 ± 8.8 Group2: 41.1 ± 7.2
Dmochowski et al., 2019 [100]	PFM	pelvic area	30 min stimulation sessions, 5 times per week	Women, INC Group 1: ES Group 2: comparator device	Group 1: 89 Group 2: 91	46.9
Terlikowski et al., 2013 [101]	PFM	Intravaginal	20 min stimulation sessions, twice a day	Women, INC Group 1: ES Group 2: CG	Group 1:64 Group 2:29	Group 1: 46.9 ± 6.8 Group 2: 45.6 ± 7.9
Elena et al., 2020 [102]	PFM	Group 1: chair Group 2: intravaginal	28 min stimulation sessions, 2–3 times per week	Women, PFD Group 1: ES1 Group 2: ES2 Group 3: healthy CG	Group 1: 50 Group 2: 25 Group 3: 20	Group 1: 31.12 ± 1.52 Group 2: 31.96 ± 3.20 Group 3: 27.20 ± 2.02
Huebner et al., 2011 [103]	PFM	Intravaginal	15 min stimulation sessions, 2 times a day	Women, INC Group 1: PFMT + ES1 Group 2: PFMT + ES2 Group 3: PFMT	Group 1: 33 Group 2: 28 Group 3: 27	49.8 ± 12.9
**ES—Dualpex 961^®^ Quark Co**						
Mateus-Vasconcelos et al., 2018 [104]	PFM	Intravaginal	20 min stimulation sessions	Women, PFD Group 1: ES Group 2–4: CG	Group 1: 33 Group 2: 33 Group 3: 33 Group 4: 33	Group 1: 55.6 ± 10.3 Group 2: 53.7 ± 14.0 Group 3: 49.6 ± 11.3 Group 4: 53.5 ± 14.0
Fürst et al., 2014 [105]	PFM	Intravaginal	30 min stimulation sessions, twice a week	Women, INC Group 1: ES Group 2: ES + PFMT	Group 1: 24 Group 2: 24	49.6 ± 10.60
Alves et al., 2011 [106]	Neuromuscular—PFM	Intravaginal	20 min stimulation sessions, twice a week	Women, INC	20	55.55 ± 6.51
Pereira et al., 2012 [107]	PFM	Suprapubic region and ischial tuberosity	20 min stimulation sessions, twice a week	Women, INC Group 1: ES Group 2: CG	Group 1: 7 Group 2: 7	Group 1: 68.57 ± 10.93 Group 2: 69.28 ± 6.94
Correia et al., 2014 [108]	PFM	Group 1: suprapubic region and ischial tuberosity Group 2: intravaginal	20 min stimulation sessions, twice a week	Women, INC Group 1: surface ES Group 2: intra-vaginal ES Group 3: CG	Group 1: 15 Group2: 15 Group3: 15	Group 1: 64.46 ± 8.83 Group2: 59.86 ± 4.82 Group3: 60.13 ± 9.35

**Table 4:** Data extracted from the reviewed studies including ultrasound.

Study	Obtained Parameter	Sensor Position	Test Activity	Subject Characteristics		
				Group	Number	Mean Age (Years)
**Ultrasound**						
Speksnijder et al., 2012 [109]	Levator ani hiatus during maximal contraction	Transabdominal	MVC; supine	Symptomatic Women	100	57
Thompson et al., 2005 [110]	PFM contraction	Transabdominal transperineal	MVC, Valsalva; supine	Women Group 1: INC Group 2: CG	Group 1: 60 Group 2: 60	43 ± 7
Dietz et al., 2001 [111]	Bladder neck displacement	Translabial	Rest, MVC; supine	Women Group 1: INC Group 2: CG	212	53.8
Yoshida et al., 2017 [112]	Bladder base displacement; PF morphology	Transabdominal	MVC; supine	Women Group 1: INC Group 2: CG	Group 1: 22 Group 2: 51	Group 1: 33.2 ± 4.7 Group 2: 32.2 ± 4.2
Thompson and O’Sullivan, 2003 [113]	Bladder and levator Plate displacement	Transabdominal	MVC; supine	Patients with INC and/or POP	104	45.03 ± 13.1
Lovegrove Jones et al., 2010 [114]	Kinematics of urethra and pubic symphysis; anorectal angle	Perineal	Coughing; supine	Women Group 1: INC Group 2: CG	Group 1: 9 Group 2: 23	Group 1: 47.9 ± 13.2 Group 2: 41.1 ± 13.6
Kruger et al., 2007 [115]	Pelvic organ displacement; levator hiatus	Translabial	Rest, MVC, Valsalva; supine	Women: Group 1: HIFIT athletes Group 2: CG	Group 1: 24 Group 2: 22	Group 1: 28.5 Group 2: 27.6
McLean et al., 2013 [116]	Urethral morphology and mobility	Transperineal	Coughing, Valsalva; supine	Women, INC Group 1: PFMT Group 2: CG	Group 1: 15 Group 2: 17	Group 1: 49.5 ± 8.2 Group 2: 54.0 ± 8.4
Oleksy et al., 2019 [117]	PFM asymmetry	Transabdominal	Rest, MVC	Healthy, nulliparous women	30	- (young)
Dietz et al., 2001 [118]	Position of the bladder neck, leading edge of a cystocele, the cervix, cul-de-sac, and rectum	Translabial	Coughing, MVC; supine	-	145	-

**Table 5:** Data extracted from the reviewed studies including magnetic stimulation.

Study	Stimulated Part	Sensor Position	Stimulation Type	Subject Characteristics		
				Group	Number	Mean Age (Years)
**Extracorporeal MS—Neo Control Chair**						
Weber-Rajek et al., 2020 [119]	Transversus abdominis muscle, perineum	Chair	Group 1: 45 min sessions, 3 times a week Group 2: 15 min sessions, 3 times a week	Women, INC Group 1: MS 1 Group 2: MS 2 Group 3: CG	Group 1: 40 Group 2: 37 Group 3: 34	Group 1: 70.12 Group 2: 66.71 Group 3: 69.79
Galloway et al., 1999 [120]	Transversus abdominis muscle, perineum	Chair	20 min sessions, twice a week	Women, INC	64	55 ± 12
Yokoyama et al., 2004 [121]	Transversus abdominis muscle, perineum	Chair	20 min sessions, twice a week	INC Group 1: stress-UI Group 2: urge UI	Group 1: 17 Group 2: 15	Group 1: 60.1 ± 12.6 Group 2: 68.5 ± 14.2
Ünsal et al., 2003 [122]	Transversus abdominis muscle, perineum	Chair	20 min sessions, twice a week	Women, INC Group 1: stress-UI Group 2: urge UI	Group 1: 29 Group 2: 15	Group 1: 55 Group 2: 58
**Extracorporeal MS—QRS^®^-1010 PelviCenter**						
Lim et al., 2018 [123]	PFM, thigh muscles, gluteus muscles, lumbar spine	Chair	20 min sessions, twice a week	Women, INC Group 1: MS Group 2: CG	Group 1: 57 Group 2: 58	Group 1: 51.8 ± 10.0 Group 2: 52.7 ± 7.8
Lim et al., 2017 [124]	PFM, thigh muscles, gluteus muscles, lumbar spine	Chair	20 min sessions, twice a week	Women, INC Group 1: MS Group 2: CG	Group 1: 57 Group 2: 58	Group 1: 51.8 ± 10.0 Group 2: 52.7 ± 7.8
**Other MS**						
Sun et al., 2015 [125]	PFM, sacral roots	Chair	20 min sessions, twice a week	women with urinary tract dysfunction following radical hysterectomy	32	Median age: 61

**Table 6:** Data extracted from the reviewed studies including MRI.

Study	Obtained Parameter	Sensor Position	Test Activity	Subject Characteristics		
				Group	Number	Mean Age (Years)
**MRI**						
DeLancey, 2003 [126]	Levator ani muscle	-	-	Women: Group 1: NP Group 2: primiparas and CON Group 3: primiparas and INC	Group 1: 80 Group 2: 80 Group 3: 80	Group 1: 29.2 ± 5.5 Group 2: 29.8 ± 4.4 Group 3: 30.0 ± 5.7
**Dynamic MRI**						
El-Gharib, 2013 [127]	pubococcygeal line	-	Supine position	Patients having clinical manifestations suggesting pelvic floor weakness	60	38 ± 4.2
Bø et al., 2001 [128]	Movement of bladder neck and coccyx movement	-	PFM function during contraction and straining in an upright sitting position	Women; Group 1: CON Group 2: INC	Group 1: 9 Group 2: 7	Group 1: 39.4 ± 3.9 Group 2: 52.4 ± 8.3
Grassi et al., 2007 [129]	Coccyx movement	-	Defecation act in supine position: rest, contraction, straining and evacuation	Women; Men; Patients with clinical indication for dynamic magnetic resonance imaging (MRI) defecography	Women: 95 Men: 17	Women: 58.0 (25–84) Men: 67.0 (33–75)
Fujisaki et al., 2018 [130]	Coccyx movement	-	PFM contraction and strain	Women; Group 1: INC Group 2: CG	Group 1: 57 Group 2: 6	median: 50 (30–81)
Talasz et al., 2011 [131]	Cranio-caudal movement of diaphragm and PF	-	Breathing, coughing in supine position	Healthy volunteers	8	25 ± 6 (19–33)

**Table 7:** Data extracted from the reviewed studies including all other sensor technology, such as X-ray, electromagnetic tracking, photogrammetry, accelerometer, vibration, and infrared thermography.

Study	Obtained Parameter	Sensor Position	Test Activity	Subject Characteristics		
				Group	Number	Mean Age (Years)
**X-ray**						
Lind et al., 1996 [132]	Thoracic kyphosis	X-ray	Standing in natural, upright postures, arms clasped overhead	Women; Group 1: advanced uterine prolapse Group 2: no evidence of prolapse	Group 1: 48 Group 2: 48	matched age (±4 years) of Group 1 and Group 2
Park and Han, 2015 [133]	Diaphragmatic motion with contraction of the PFM during breathing	X-ray	Diaphragmatic motion was measured before and during contraction of the PFM in a supine position	Healthy women	20	22.5
Nguyen et al., 2000 [134]	Angles of lumbar lordosis and pelvic inlet	X-ray	Participants standing in their usual upright posture, with their shoes on and their hands at chest level	Parous women; Group 1: with prolapse Group 2: without prolapse	Group 1: 20 Group 2: 20	Group 1: 55.3 ± 9.0 Group 2: 53.4 ± 9.5
**EMT**						
Leitner et al., 2018 [135]	PFM kinematics	Sensor 1: vaginal probe Sensor 2: skin, second sacral vertebrae	Running at speeds of 7, 11, and 15 km/h	Women; Group 1: CON Group 2: INC	Group 1: 27 Group 2: 19	Group 1: 38.7 (18–60) Group 2: 45.3 (18–60)
Moser et al., 2019 [136]	PFM kinematics	Sensor 1: vaginal probe Sensor 2: skin, second sacral vertebrae	CMJ and DJ	Women; Group 1: CON Group 2: INC	Group 1: 24 Group 2: 21	Group 1: 39.3 (18–60) Group 2: 45.8 (18–60)
**Photogrammetry**						
Szczygieł et al., 2018 [137]	Posture (head, pelvic, trunk) and breathing	Reflective markers on head, pelvic, and trunk	Different exercises	Participants without any respiratory disorders, chest deformations, pain complaints, or visible postural defects	18	25.7 ± 3.5
Zhoolideh et al., 2021 [138]	Lumbar lordosis and thoracic kyphosis, head sagittal tilt angle, head coronal tilt, scapular alignment	Reflex markers on anatomical landmarks	-	Women; Group 1: with PFDs Group 2: CG	Group 1: 47 Group 2: 47	Group 1: 37.74 ± 6.29 Group 2: 37.43 ± 6.17
**Accelerometer**						
Niederauer et al., 2022 [139]	Intravaginal acceleration, pelvis acceleration	Posterior fornix of the vagina	Running at different speeds	Women; Group 1: CON Group 2: INC	Group 1: 7 Group 2: 10	Group 1: 39.8 ± 11.3 Group 2: 45.6 ± 11.8
Bohorquez et al., 2020 [140]	Vaginal tilt angle, fornix tilt angle	Anterior, posterior, and lateral areas of the fornix	Rest, Valsalva, MVC, with maximal hold, during repeated contractions in supine, seated and standing position	Women with INC	10	>18
**Vibration**						
Lauper et al., 2009 [141]	-	Whole-body vibration platform	MVC, different vibration intensities, vibration + MVC	Women; Group 1: Post-partum Group 2: CG	Group 1: 17 Group 2: 21	Group 1: 31.7 ± 3.4 Group 2: 30.0 ± 4.7
**IRT**						
Da Silva et al., 2022 [142]	PF temperature	Camera perpendicular to the perineum	Rest, MVC in supine position (with bent knees and flexed and abducted hips)	Women;	231	58.4 ± 6

**Table 8:** The most common factors in the analyzed studies, with a color-coded classification of each parameter according to the level of evidence stated in the respective study. Green color represents statistical correlation between the factor and the pelvic floor health. Red color means that no correlation was found.

	Factors	Age	Menopause	Parity	Mode of Delivery	Fetal Macrosomia	Hormone Therapy	BMI	Race	Chronic Cough	Hysterectomy	Physical Activity	Gastrointestinal Pathology	Gynecological Pathology	Other Diseases *	Respiration	Spine Curvature
Study																	
Kim et al., 2007 [143]																	
Peinado-Molina et al., 2023 [144]																	
Kepenekci et al., 2011 [145]																	
Eftekhar et al., 2012 [146]																	
Nygaard et al., 1994 [147]																	
MacLennan et al., 2000 [1]																	
Wu et al., 2014 [148]																	
Nygaard et al., 2008 [14]																	
Hwang et al., 2021 [99]																	
Vieira et al., 2020 [149]																	
Rotveit et al., 2001 [150]																	
Wasserberg et al., 2007 [151]																	
Capson et al., 2011 [90]																	
Mattox et al., 2000 [152]																	
Melli and Alizadeh, 2007 [153]																	
Nguyen et al., 2000 [134]																	
Zhoolideh et al., 2021 [138]																	

* Arthritis and osteoporosis.

## Supplementary Materials

The following supporting information can be downloaded at https://www.mdpi.com/article/10.3390/s24124001/s1, Table S1: Extended results table.

## List of Abbreviations

The following additional abbreviations were used in the final results tables.

AMM	abdominal muscle maneuver
BF	biofeedback
CG	control group
CMJ	counter movement jump
CON	continence
CPP	chronic pelvic pain
DJ	drop jumps
INC	incontinence
MA	muscle activity
MEC	maximal endurance contraction
MVC	maximal voluntary contraction
NP	nulliparous
P	parous
PFME	pelvic floor muscle exercise
PP	perineal pressure
